# Comparison of MRI cross-sectional area and functions of core muscles among asymptomatic individuals with and without lumbar intervertebral disc degeneration

**DOI:** 10.1186/s12891-019-2960-y

**Published:** 2019-12-01

**Authors:** Gyeong-tae Gwak, Ui-jae Hwang, Sung-hoon Jung, Hyun-a Kim, Jun-hee Kim, Oh-yun Kwon

**Affiliations:** 1 Department of Physical Therapy, Graduate School, Yonsei University, Wonju, Republic of Korea; 2 Department of Physical Therapy, College of Health Science, Laboratory of Kinetic Ergocise Based on Movement Analysis, Yonsei University, Wonju, Republic of Korea

**Keywords:** Asymptomatic diseases, Intervertebral disc degeneration, MRI, Cross-sectional area, Muscle functions, Core muscles

## Abstract

**Background:**

Previous studies suggested that patients with symptomatic intervertebral disc degeneration (IDD) of lumbar spine have reduced cross-sectional area (CSA) and functions of core muscles. However, reduced CSA and functions of core muscles have been observed not only in patients with symptomatic IDD but also in patients with other subgroups of low back pain (LBP). Thus, it is uncertain whether reduced CSA and functions of core muscles lead to IDD and LBP, or pain leads to reduced CSA and functions of core muscles in patients with symptomatic IDD. Therefore, this study aimed to compare the CSA and functions of core muscles between asymptomatic participants with and without IDD in magnetic resonance imaging (MRI).

**Methods:**

Twenty asymptomatic participants (12 men and 8 women) participated in this study. Ten participants had asymptomatic IDD at L4–5. The others were healthy controls (without IDD at all levels of lumbar spine). The CSA of core muscles was measured using MRI. Maximal isometric trunk flexor strength and side bridge strength were measured by a Smart KEMA strength sensor. Trunk flexor endurance test, side bridge endurance test and plank endurance test were used to measure core endurance. Double legs loading test was used to measure core stability. Mann-Whitney U test was used to compare the differences between two groups.

**Results:**

There were no significant differences in core muscle functions between the two groups (*p* > 0.05). Moreover, there was no significant difference in CSA between the two groups (p > 0.05).

**Conclusions:**

There was no significant difference in CSA and core muscle functions between asymptomatic participants with and without IDD. These findings indicate that a degenerative or bulging disc in asymptomatic individuals has little effect on CSA and functions of core muscles, especially in young age. Therefore, the general core endurance test or strength test could not differentiate asymptomatic people with and without IDD of lumbar spine.

**Trial registration number:**

Clinical Research information Service. KCT0004061. Registered 13 June 2019. retrospectively registered.

## Background

Intervertebral disc degeneration (IDD) of the lumbar spine is an underlying factor of low back pain (LBP) [1]. Magnetic resonance imaging (MRI) is the most widely used method for clinical assessment of IDD of the lumbar spine [2, 3]. Loss of water content, proteoglycans, and collagens that occur in IDD can be visualized on MRI under T2 weighting with a hypointense signal [2]. With the loss of proteoglycans, the osmotic pressure of the disc falls, and the disc cannot maintain hydration under load [4]. Consequently, IDD alters disc height and the mechanics of the rest of the spinal column, possibly adversely affecting the behavior of other spinal structures such as muscles and ligaments [5].

Biomechanical studies suggested that buckling failure of the lumbar spine, devoid of muscle, occurs with compressive loading of as little as 90 N [6, 7]. Thus, muscle activity is required to stiffen intervertebral joints, and muscle functions (strength and endurance) are required to meet the demands of control [6, 7]. Core muscles comprise a muscular box with the abdominals (rectus abdominis, external oblique, internal oblique, transversus abdominis) in the front, para-spinal muscles (erector spinae, multifidus, quadratus lumborum, psoas major) in the back, diaphragm as the roof, and pelvic floor musculature as the bottom around the spine [8]. Core muscles serve as a muscular corset that stabilizes the body and spine [9]. Therefore, isometric core muscle endurance tests, plank endurance test and double-leg loading test have been used to measure the core muscle functions in clinical and field setting [10–14]. The cross-sectional area (CSA) of core muscles has been associated with the capacity of muscles to generate force [15], as reduced CSA of the core muscles could lead to muscle force imbalance [16]. Muscle force imbalance may lead to kinetic instability of the spine, a possible reason for LBP [16].

Previous studies investigating the differences between patients with symptomatic IDD of lumbar spine and healthy controls showed that these patients have reduced CSA of paraspinal muscles [17–22], which is supported by laboratory studies [23–25]. Experimentally induced disc or nerve injury in animals showed rapid atrophy of paraspinal muscles [23–25]. However, reduced CSA of the paraspinal muscles and functions of core muscles have been observed not only in patients with symptomatic IDD but also in patients with other subgroups of LBP [16, 26, 27]. In patients with LBP, pain could be responsible for the reduced CSA and core muscle function [27], causing disuse atrophy [28] and reflexive inhibition [26]. Induced pain on paraspinal muscles after hypertonic saline injections could cause reduced muscle activity [24, 25]. Thus, it is uncertain whether reduced CSA and functions of core muscles lead to IDD and LBP, or pain leads to reduced CSA and functions of core muscles in patients with symptomatic IDD. Therefore, this study aimed to compare the CSA and functions of core muscles between asymptomatic participants with and without IDD in MRI.

## Methods

### Participants

**Fig. 1:**
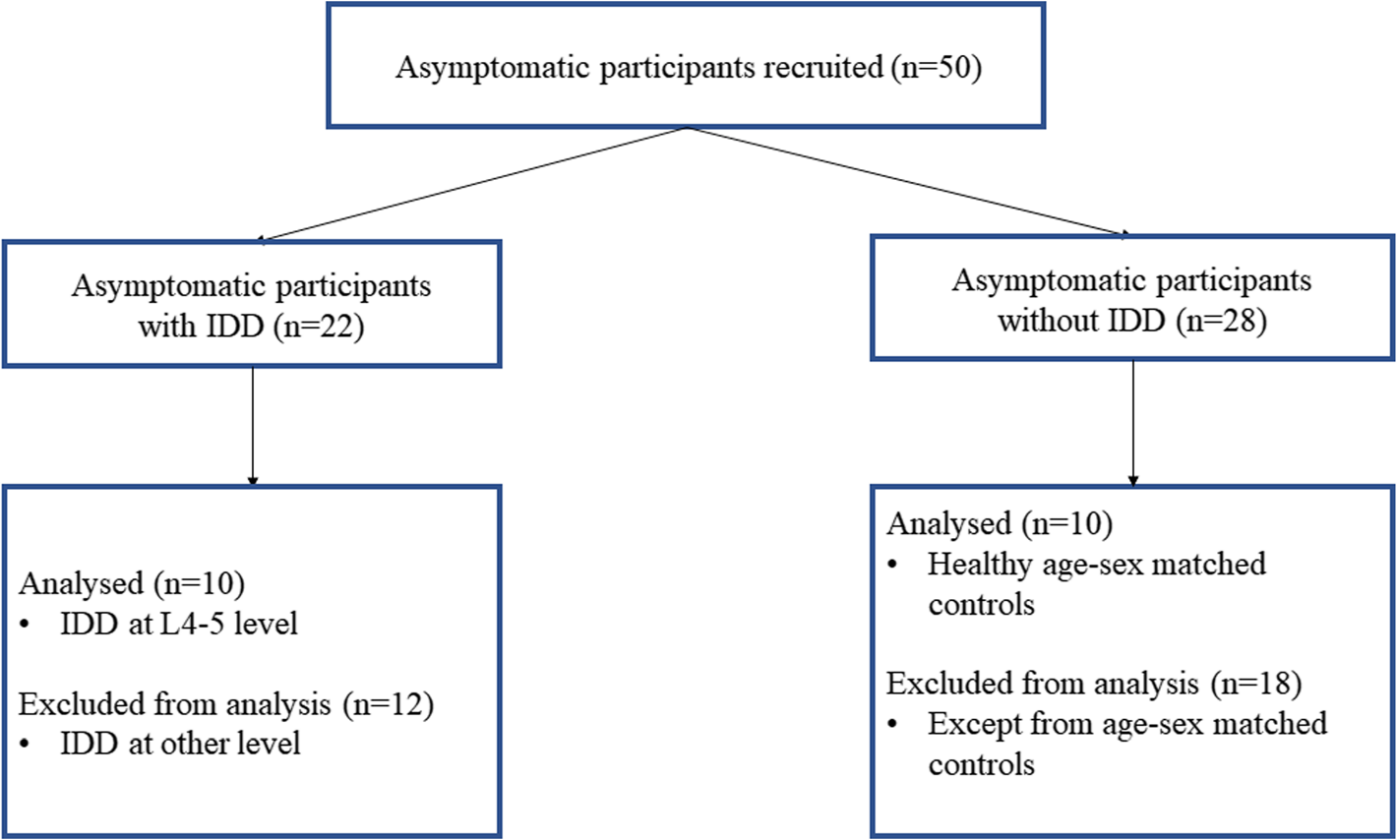
Flowchart showing a schematic summary of participants recruitment and data analysis in this study

Fifty asymptomatic participants (25 men and 25 women) recruited to the study. Twenty two of fifty participants had asymptomatic IDD (at the level of L3–4 in 1 participant, at L4–5 in 10 participants, at L5-S1 in 11 participants). Twenty of fifty asymptomatic participants (12 men and 8 women) participated in this study (Fig. 1). Of asymptomatic participants with IDD, only those who had asymptomatic IDD at L4–5 level were participated in this study (*n* = 10) due to limitation of analytical methods. The others were healthy matched controls for age, sex and weight (without IDD at all levels of lumbar spine). Participants were included if they did not have current LBP or a history of back pain in the last 12 months, previous spinal surgery or spinal fracture, neurological or orthopedic disease, or open abdominal surgery [29]. Participants were excluded if they had contraindications to MRI such as metallic implants, cardiac pacemakers, or claustrophobia [30]. Before the study, all participants received explanations about all procedures of the study and signed an informed consent form approved by the Institutional Review Board of Yonsei University Wonju Institutional Review Board (1041849–201,904-BM-053-01).

### Magnetic resonance imaging assessment

**Fig. 2:**
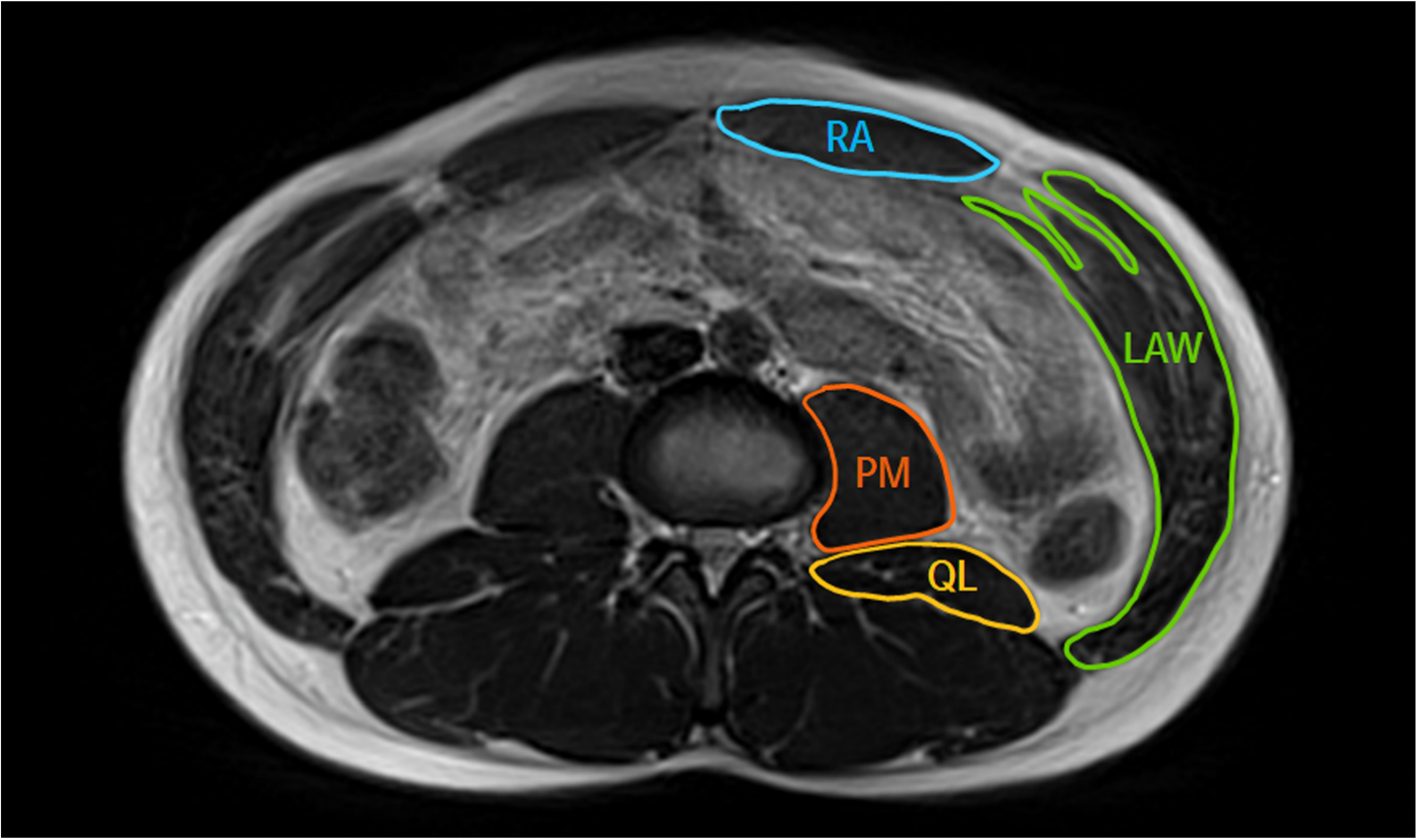
Magnetic resonance imaging of abdominal muscles and paraspinal muscles at L3–4 level. RA = Rectus abdominis; LAW = Lateral abdominal wall; QL = Quadratus lumborum; PM = Psoas major

**Fig. 3:**
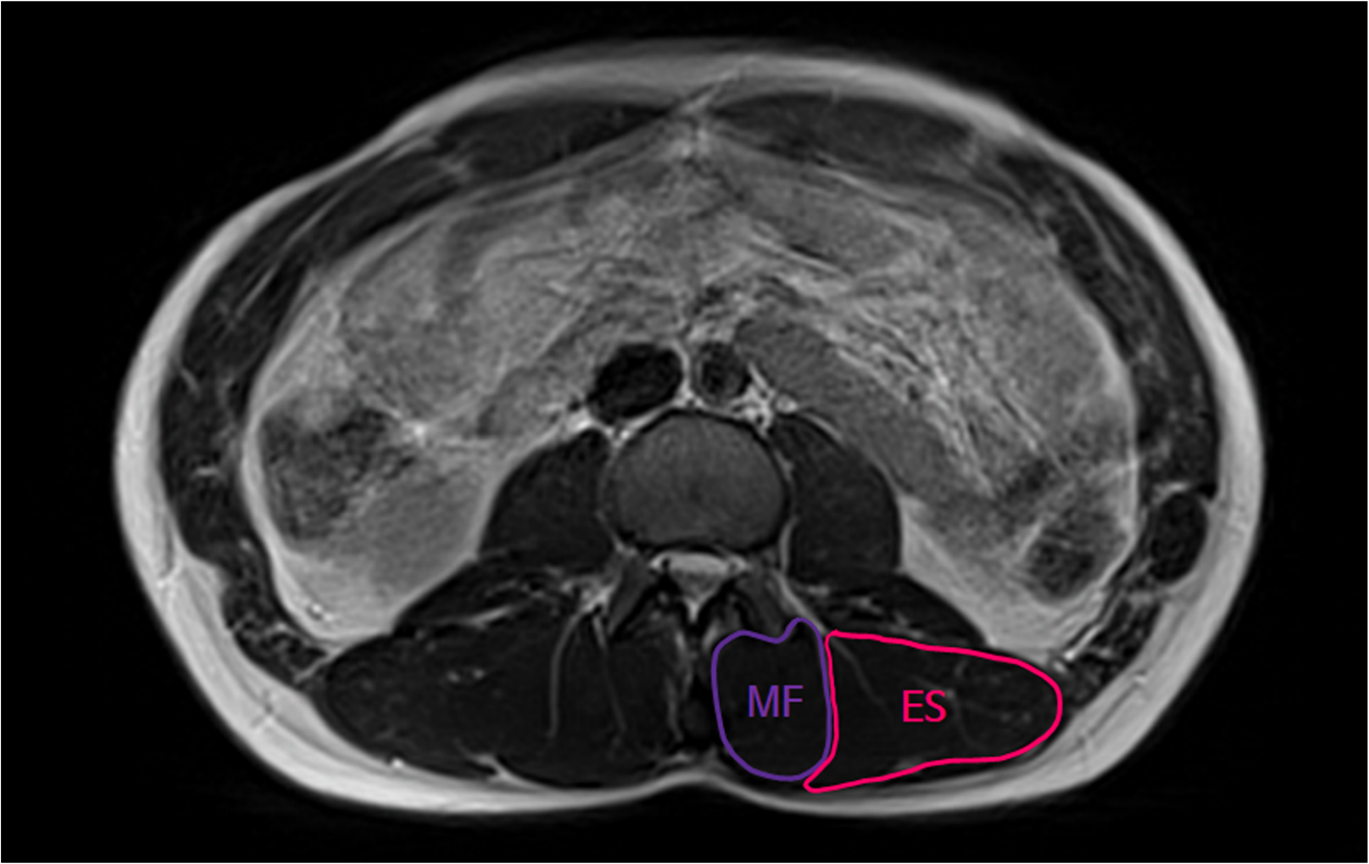
Magnetic resonance imaging of erector spinae and multifidus muscles at L4 vertebral endplate. ES = Erector spinae; MF = Multifidus

MR scans were obtained using a 1.5 T Magnetom Avanto (Siemens, Erlangen, Germany). T2 axial MR scans were obtained using repetition time (TR)/echo time (TE) = 2100 ms/91 ms, 256 × 156 matrix, field of view (FOV) 400 × 325 mm, and slice thickness 5 mm. Participants were required to hold their breath during the MR scan to prevent movement artifacts. MR scans were analyzed using the PACSPLUS Viewer (Medical Standard, Seongnam, Korea). Based on the Pfirrmann classification, the discs were then divided into two groups: without IDD at all levels of lumbar spine (Pfirrmann ≤2) and with IDD at L4–5 level (Pfirrmann ≥3) [31]. The CSA of abdominal muscles (rectus abdominis, lateral abdominal wall) and paraspinal muscles (erector spinae, multifidus, quadratus lumborum, psoas major) were measured by free-hand drawing using the PACSPLUS Viewer in both groups. The CSA of abdominal muscles and paraspinal muscles, except for the multifidus, was measured using the MR images centered on L3–4 (Fig. 2). Considering nerve innervation of the multifidus, the CSA of multifidus was measured using the MR images centered on the L4 lower vertebral endplate (Fig. 3) [32]. All data analysis was conducted by one assessor for consistency.

### Measurement of maximal isometric trunk flexor and side bridge strength

**Fig. 4:**
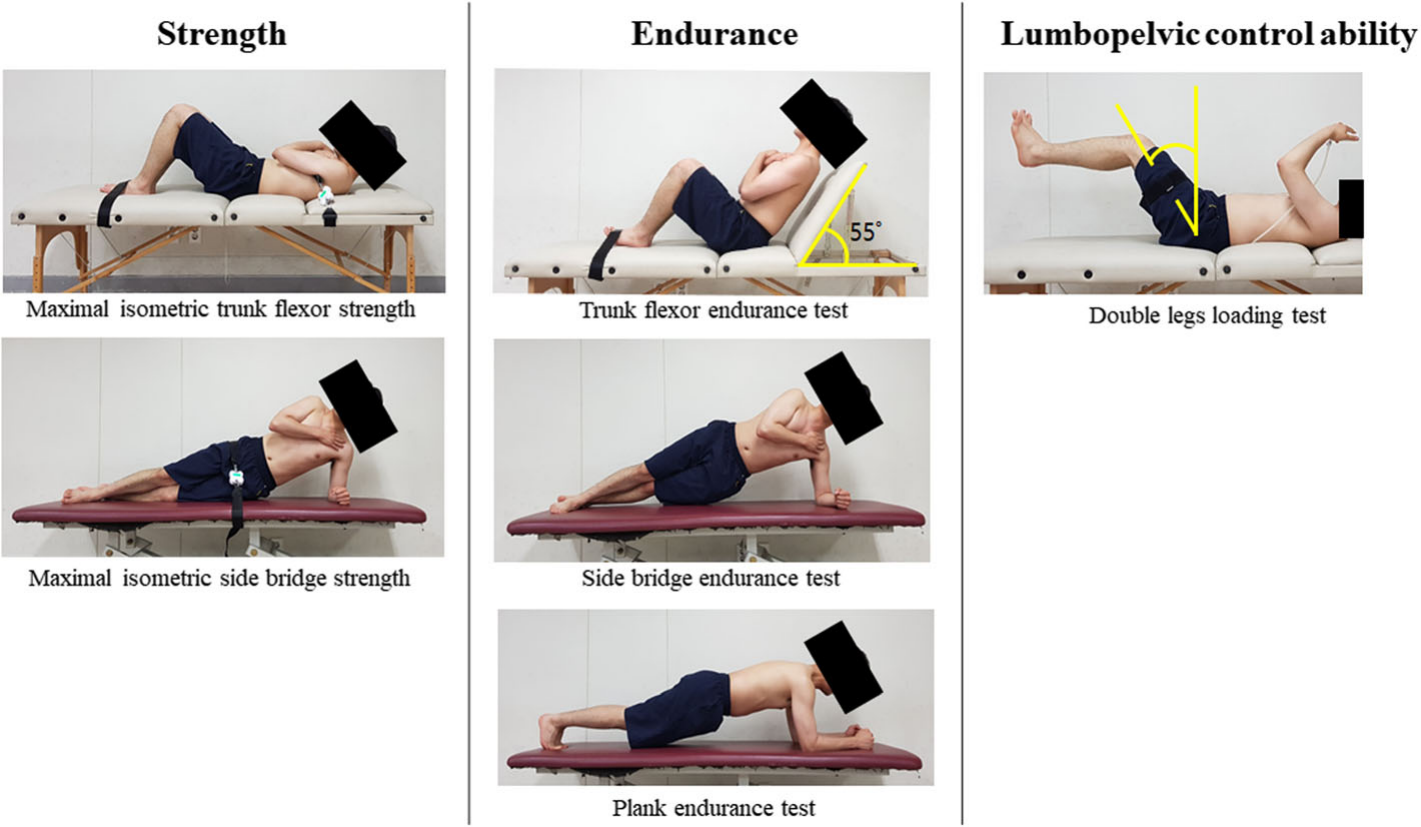
Core muscles function test

Maximal isometric trunk flexor strength and side bridge strength were measured by a Smart KEMA strength sensor (KOREATECH, Inc., Seoul, Korea) [33, 34]. Data measured using the sensor were transferred to a tablet PC (Galaxy Tab A6 10.1; Samsung, Inc., Seoul, Korea) via bluetooth network and analyzed using the Smart KEMA application (KOREATECH, Inc., Seoul, Korea). To measure maximal isometric trunk flexor strength [35], participants laid in hook lying position on a table with their feet fixed to the table with an adjustable belt to stabilize the lower limbs during trunk flexion. A belt was connected to the sensor placed on the middle of the sternum. Participants were instructed to sit up and pull the belt with maximum efforts. While participants performed trunk flexion maximally, the sensor measured the maximal isometric strength as both sides of the sensor were pulled by the belts (Fig. 4). To measure maximal isometric side bridge strength [36], the participants laid on their sides with their legs extended. A belt connected to the strength sensor was placed between the top of the iliac crest and the greater trochanter. Participants were instructed to lift their hips off the table and to rest on one elbow and their feet with maximum effort. While participants performed the side bridge task, the sensor measured the maximal isometric strength (Fig. 4). The initial tension value for measuring maximal isometric strength was set to 2 kgf in the start position to control the tension [34]. Both sides were measured and the sum value of both sides was used for data analysis. Participants were asked to maintain maximal isometric strength for 5 s to reach the maximal strength plateau [37], and the average value was calculated from the middle 3 s to eliminate the effect of insufficient muscle recruitment during first 1 s and the effect of muscle fatigue during last 1 s. The mean values of two trials were used for data analyses. There was a 30-s rest after each trial and a 5-min rest between conditions to prevent muscle fatigue.

### Measurement of core muscle endurance

To test the trunk flexor endurance, the participants held a sit-up posture with their back resting against back supports angled 55° away from the table (Fig. 4) [10, 13]. Both knees and hips were flexed to 90°. The arms were folded across their chest with their hands placed on the opposite shoulders, and their feet were placed under feet straps. The participants were instructed to maintain the body position while the back supports were pulled back 10 cm to begin the test according to previous reported methods [10, 13]. Failure is determined to occur when any part of the person’s back touched the back supports, or the participant requested to stop, or if they were unable to preserve form after one verbal warning. To test the lateral musculature endurance, the side bridge endurance test was performed (Fig. 4) [10, 13]. The participants lay on their sides with the legs extended. The top foot was placed in front of the lower foot for support. Participants were instructed to maintain support on one elbow and on their feet while lifting their hips off the floor to maintain a straight line over their body length. The uninvolved arm was held across the chest with the hand placed on the opposite shoulder. The test ended when the participants lost the straight-back posture and the hip returned to the ground, the participants requested to stop, or they were unable to preserve form after one verbal warning. Both sides were measured, and the positive value of both sides was used for data analysis. For the plank endurance test (Fig. 4) [12, 38], participants lay prone with the elbows in contact with the ground, such that the humerus formed a perpendicular line with the horizontal plane, directly beneath the shoulders. The elbows were spaced shoulder-width apart in a neutral position, and the feet were set with a narrow base, but not touching. The participants were then instructed to raise their pelvis from the floor so that only the forearms and toes were in contact with the floor while maintaining their shoulders, hips, and ankles in a straight line. The test ended when the straight line was no longer be maintained, and the hips dropped toward the floor, or the participants requested to stop, or they were unable to preserve form after one verbal warning. During the endurance tests, participants were reminded to maintain the position for as long as possible. To avoid muscle fatigue, participants rested for 5 min between conditions.

### Measurement of lumbopelvic control ability (modified double legs loading test)

To test the ability to control the spine, the modified double legs loading test was performed by maintaining abdominal pressure during movements of the lower limbs (Fig. 4) [14, 39]. The participants were in the supine with their hip and knee flexed to 90°. A Smart KEMA pressure sensor (KOREATECH, Inc.) was set to 40 mmHg and placed below the lordotic curvature of the spine between S1 and L1 with the hip and knee in 90° flexion. A strap with a Smart KEMA motion sensor (KOREATECH, Inc.) was placed on the lateral thigh between the greater trochanter and knee joint. Participants were instructed to maintain the 40 mmHg pressure while extending both legs. The angle of both legs lowering (hip extension) was measured with a motion sensor was defined as lumbopelvic control ability and when pressure changed more than 10 mmHg during both-legs lowering. The mean values of two trials were used for data analyses for both legs lowering. To avoid muscle fatigue, participants rested for 5 min between tests.

### Statistical analyses

SPSS ver. 25.0 (SPSS, Inc., Chicago, IL, USA) was used for statistical analysis. The ICC (3, 1) model was used to test intra-rater reliability of measurements. Data normality was examined using the Shapiro-Wilk test. Medians and interquartile ranges (25th–75th percentile) were used for descriptive analyses of quantitative variables due to significance of some variables. Therefore, non-parametric tests were deemed more adequate for statistical analysis. Comparisons were performed using the Mann-Whitney U test. The level of statistical significance was set at α = 0.05. The software G*Power 3.1.9.2 was used to estimate the sample size needed for the study. A power analysis based on the mean and standard deviation from the study by Abdelraouf and Abdel-Aziem [40] demonstrated that at least 8 participants were required in each group with 80% power and alpha value of 0.05.

## Results

The general characteristics of participants are listed in Table 1. There were no significant differences in age, height, or body weight between the two groups. Among 10 cases of IDD, 9 were grade 3 and 1 was grade 4. There were no significant differences in core muscle functions between the two groups (*p* > 0.05) (Table 2). The ICC (3, 1) for measuring trunk flexor strength was 0.967 [95% CI 0.918–0.987], for side bridge strength was 0.959 [95% CI 0.901–0.984], and for the modified double legs loading test was 0.981 [95% CI 0.952–0.992]. Moreover, there was no significant difference in CSA between the two groups (p > 0.05) (Table 3).

**Table 1:** Characteristics of study participants

Variables	With IDD	Without IDD	*p*-value
Age (years)	25.00 (23.25/25.25)	24.50 (23.00/28.75)	0.739
Height (cm)	173.50 (164.50/176.00)	175.00 (159.50/176.93)	0.631
Weight (kg)	69.40 (55.63/78.55)	70.85 (53.43/78.88)	0.912

Data is presented as median (IQR 25th/75th)

*IDD* Intervertebral disc degeneration

**Table 2:** Comparison of functions of core muscles between two groups

Variables	With IDD	Without IDD	p-value
Trunk flexor strength test (kgf)	20.10 (11.79/37.19)	18.88 (11.54/27.00)	0.631
Side bridge strength (kgf)	44.53 (25.35/61.34)	32.58 (22.93/52.75)	0.481
Trunk flexor endurance test (s)	92.05 (58.60/164.58)	104.42 (53.04/143.51)	0.631
Side bridge endurance test (s)	108.54 (67.07/134.84)	103.19 (76.82/134.70)	0.853
Plank endurance test (s)	64.07 (44.37/87.47)	56.07 (44.88/75.39)	0.684
Modified double legs loading test (°)	22.98 (9.64/37.54)	23.75 (10.16/62.36)	0.912

Data is presented as median (IQR 25th/75th)

*IDD* Intervertebral disc degeneration

**Table 3:** Comparison of cross-sectional area of core muscles between two groups

Variables	With IDD	Without IDD	p-value
Rectus abdominis (cm^2^)	10.73 (7.39/13.60)	10.11 (8.11/14.50)	0.739
Lateral abdominal wall (cm^2^)	52.70 (28.56/60.47)	42.43 (30.68/49.12)	0.353
Erector spinae (cm^2^)	20.91 (15.48/30.66)	20.97 (16.67/25.34)	0.796
Multifidus (cm^2^)	12.18 (9.20/15.59)	10.64 (9.45/12.50)	0.579
Quadratus lumborum (cm^2^)	12.05 (5.50/14.96)	12.48 (6.90/14.02)	0.912
Iliopsoas (cm^2^)	22.81 (12.50/29.77)	24.96 (12.30/26.17)	0.631

Data is presented as median (IQR 25th/75th)

*IDD* Intervertebral disc degeneration

## Discussion

Patients with LBP or symptomatic IDD of lumbar spine have reduced CSA of paraspinal muscles and core muscle function compared to healthy controls [17–22]. However, in this study, we investigated the difference in CSA and core muscle functions between asymptomatic participants with and without IDD. There was no difference in CSA and core muscle function between the two groups. There are possible reasons for the lack of differences between the two groups.

In previous studies [17–22], because participants were patients with LBP or symptomatic IDD of lumbar spine, pain could be responsible for the reduced CSA and core muscle functions [27]. The effect of pain on muscle function, reducing the ability to contract, was demonstrated experimentally using a model of induced pain [25, 41]. Similarly, patients with LBP reportedly have a reduction in the ability to voluntarily contract the multifidus muscle [27]. In addition to muscle inhibition caused by pain, disuse and deconditioning due to pain could induce disuse muscle atrophy [42]. Because the CSA of muscles has been associated with the muscle’s capacity to generate force, reduced CSA of core muscles could decrease the function of core muscles [42]. In this study, participants were asymptomatic with and without IDD of the lumbar spine. It would be one possible reason why there was no significant difference in CSA and functions of core muscles between the groups.

The compression caused by the herniated disc on the dorsal and/or the ventral nerve roots is believed to cause LBP, sciatica, muscle spasms, and restriction of trunk movement [17]. Ploumis et al. reported ipsilateral atrophy of the paraspinal muscles in patients with unilateral back pain with monosegmental degenerative disc disease [21]. The mechanism of reduced CSA of ipsilateral paraspinal muscles may not be generalized disuse atrophy or spinal reflex inhibition [43]. Experimentally induced disc or nerve injury in animals showed rapid atrophy (3 days postinjury) of the paraspinal muscles [23–25]. IDD and atrophy of the multifidus muscles may be positively correlated at the L3-L4 disc level in patients with symptomatic IDD [19]. Additionally, there was more severe atrophy of the multifidus muscles compared to patients with non-specific LBP in the study [19]. In this study, participants with IDD of the lumbar spine were rated from grade 1–5 using the Pfirrmann classification [44]. Nine participants were grade III, and one participant was grade IV IDD. Grade III IDD showed an intermediate gray signal intensity with a normal or slightly decreased disc height [44]. Disc bulging reportedly increases with the severity of disc degeneration [45]. In this study, considering that most participants with IDD were asymptomatic and grade III IDD, there might be little effect of nerve compression. A high prevalence (20–76%) of lumbar disc abnormalities has also been detected in asymptomatic individuals by MRI [46–48]. A bulging disc is also often observed in asymptomatic individuals [49]. Furthermore, the cohort studies showed that the findings on MRI were not predictive of the development or duration of LBP [50, 51]. Therefore, mere disc displacement does not appear to directly account for the pain [52]. Without pain, the effect of only IDD of the lumbar spine, not herniated, might be little on CSA and core muscle functions. This was another reason for the lack of differences between the groups.

Previous studies showed that high prevalence of IDD in young adults [47, 53–55]. Alyas et al. reported that 39.4% incidence of disc degeneration in 33 asymptomatic elite adolescent tennis players [54]. Elliott et al. reported a 21% incidence of disc degeneration or herniation in 24 male fast bowlers in cricket, who were bowling competitively freely, with a mean age of 13.7 years at a school and club level [53]. There was also no difference in the number of sit-up scores in 60 s between young fast bowlers with and without IDD in the study, showing 40 ± 7.5 and 34.5 ± 7 sit-ups scores, respectively [53]. These study results were consistent with those of our study. The results of our study showed that there was no difference in CSA and core muscle functions between young asymptomatic participants with and without IDD. This might be because there were no functional limitations and participation restrictions in young age [53–55]. Also, CSA and core muscle functions did not appear to be a causative factor of asymptomatic IDD in the lumbar spine. Therefore, clinically CSA and core muscle functions are not critical factors for evaluation or differentiation between asymptomatic individuals with and without IDD in young age. Thus, other etiological factors should be considered in young participants with asymptomatic IDD rather than CSA and core muscle function such as genetics [56], spinal alignment [31], and high mechanical stress [57].

Several limitations to the current study exist. First, the sample size is small. Thus, further study is needed to generalize the experimental results. Second, only young participants were recruited. Third, while CSA of the core muscles was measured, consistency changes in the muscle (fatty deposits or fibrous/connective tissue infiltration) were not assessed. Future research will need to investigate other morphologic and functional factors of the core muscles in symptomatic and asymptomatic participants with IDD of various ages.

## Conclusion

There was no significant difference in CSA and core muscle functions between asymptomatic participants with and without IDD. These findings indicate that a degenerative or bulging disc in asymptomatic individuals has little effect on CSA and functions of core muscles, especially in young participants. Therefore, the general core endurance test or strength test could not differentiate asymptomatic people with and without IDD of lumbar spine.

## Data Availability

The datasets generated and/or analyzed during the current study are available from the corresponding author on reasonable request.

## Abbreviations

CSA: Cross-sectional area
IDD: Intervertebral disc degeneration
LBP: Low back pain
MRI: Magnetic resonance imaging
